# Supplementary material for: Broad Whitefish (Coregonus nasus) isotopic niches: Stable isotopes reveal diverse foraging strategies and habitat use in Arctic Alaska
Source: PLoS One. 2022 Jul 26;17(7):e0270474. doi: 10.1371/journal.pone.0270474 (PMC9321764; doi:10.1371/journal.pone.0270474)
Supplement: S2 Table — Table displaying δ15N and δ13C for various Arctic plants invertebrates, fish, and mammals. (DOCX) [file pone.0270474.s002.docx]

**S2 Table**. **Summary of isotope data from Arctic ecosystems used in Fig 2.** Table displaying δ^15^N and δ^13^C for various Arctic plants, invertebrates, fish, and mammals.

|  |  |  |  |  |  |  |
| --- | --- | --- | --- | --- | --- | --- |
| **Marine Species** | **δ^15^N vs air** | **S.D.** | **δ^13^C vs VPDB** | **S.D.** | **C:N** | **S.D.** |
| Polar Cod (*Boreogadus saida*) | 15.2 | 0.7 | -18.9 | 1.0 | NA | NA |
| Colville Pink Salmon (*Oncorhynchus gorbuscha*) | 10.8 | 0.6 | -20.7 | 0.5 | NA | NA |
| Copepods (calanoid) | 9.2 | 0.5 | -20.4 | 0.4 | NA | NA |
| Particulate organic matter (POM) | 5.4 | 0.8 | -21.6 | 0.3 | NA | NA |
| Kelp (*Laminariales spp.*) | 7.1 | 1.3 | -20.1 | 0.3 | NA | NA |
| Polar bear (*Ursus maritimus*) | 21.1 | 0.6 | -18.0 | 0.6 | NA | NA |
| Source: Hodson and Welch 1992; Leppi et al. 2022 (This study) |  |  |  |  |  |  |
| **Lagoon Species** | **δ^15^N vs air** | **S.E.** | **δ^13^C vs VPDB** | **S.E.** | **C:N** | **S.E.** |
| Arctic Cisco (*Coregonus autumnalis*) | 11.5 | 1.1 | -23.0 | 0.5 | 4.3 | 0.1 |
| Copepods (calanoid) | 9.8 | 0.2 | -24.7 | 0.3 | 6.2 | 0.4 |
| Bivalves (*Macoma calcarea*) | 7.7 | 0.2 | -24.0 | 0.3 | 7.6 | 0.4 |
| Benthic particulate organic matter (BPOM) | 3.3 | 0.8 | -26.1 | 0.4 | NA | NA |
| Source: Dutton et al. 2012 |  |  |  |  |  |  |
| **Riverine species** | **δ^15^N vs air** | **S.D.** | **δ^13^C vs VPDB** | **S.D.** | **C:N** | **S.D.** |
| Amiphipod | 7.0 | NA | -24.0 | NA | NA | NA |
| Colville Northern Pike (*Esox lucius*) | 121 | 1.7 | -28.4 | 1.5 | NA | NA |
| BPOM | 1.7 | 0.2 | -25.6 | 0.2 | NA | NA |
| Umiat invertebrates | 2.3 | NA | -32.0 | NA | 5.1 | NA |
| Umiat invertebrates | 4.8 | NA | -28.0 | NA | 3.8 | NA |
| Umiat invertebrates | 2.4 | NA | -32.7 | NA | 4.7 | NA |
| Umiat invertebrates | BELOW LOQ | NA | -29.8 | NA | 8.5 | NA |
| Puviksuk invertebrates | 3.9 | NA | -27.0 | NA | 4.5 | NA |
| Colville Broad Whitefish | 9.5 | 1.32 | -26.5 | 2.3 | 3.4 | 0.0 |
| Source: Hesslein et al. 1991; Leppi et al. 2022 (This study) |  |  |  |  |  |  |
| **Lacustrine species** | **δ^15^N vs air** | **S.D.** | **δ^13^C vs VPDB** | **S.D.** | **C:N** | **S.D.** |
| Arctic Char (*Salvelinus alpinus*) | 11.9 | 5.2 | -29.6 | 1.4 | NA | NA |
| Amphipod | 5.8 | 0.7 | -31.1 | 0.8 | NA | NA |
| POM | 2.6 | 0.6 | -29.87 | 2.0 | NA | NA |
| Source: Kling et al. 1992 |  |  |  |  |  |  |
|  |  |  |  |  |  |  |
|  |  |  |  |  |  |  |
|  |  |  |  |  |  |  |

**References**

Dunton KH, Schonberg SV, Cooper LW. Food web structure of the Alaskan nearshore shelf and estuarine lagoons of the Beaufort Sea. Estuaries and Coasts. 2012; 416–435. https://doi.org/10.1007/s12237-012-9475-1

Hobson KA, Welch HE. Determination of trophic relationships within a high Arctic marine food web using δ^13^C and δ^15^N analysis. Mar Ecol Prog Ser. Inter-Research Science Center; 1992; 9–18. Available at: chrome-extension://efaidnbmnnnibpcajpcglclefindmkaj/https://www.int-res.com/articles/meps/84/m084p009.pdf

Hesslein RH, Capel MJ, Fox DE, Hallard KA. Stable isotopes of sulfur, carbon, and nitrogen as indicators of trophic level, and fish migration in the lower Mackenzie River basin, Canada. Canadian Journal of Fisheries and Aquatic Sciences.1991; 2258–2265. https://doi.org/10.1139/f91-265

Kling GW, Fry B, O’Brien WJ. Stable isotopes and planktonic trophic structure in Arctic lakes. Ecology.1992; 561–566. https://doi.org/10.2307/1940762
