# Supplementary material for: Broad Whitefish (Coregonus nasus) isotopic niches: Stable isotopes reveal diverse foraging strategies and habitat use in Arctic Alaska
Source: PLoS One. 2022 Jul 26;17(7):e0270474. doi: 10.1371/journal.pone.0270474 (PMC9321764; doi:10.1371/journal.pone.0270474)
Supplement: S3 Table — Summary of muscle tissue δD and δ18O for Pink Salmon (Oncorhynchus gorbuscha) and Northern Pike (Esox lucius) caught in the lower Colville River, AK, USA. (DOCX) [file pone.0270474.s003.docx]

**S3 Table**. **Summary of isotope data for additional fish species sampled.** Summary of muscle tissue δD and δ^18^O for Pink Salmon (*Oncorhynchus gorbuscha*) and Northern Pike (*Esox lucius*) caught in the lower Colville River, AK, USA.
