# Supplementary material for: Broad Whitefish (Coregonus nasus) isotopic niches: Stable isotopes reveal diverse foraging strategies and habitat use in Arctic Alaska
Source: PLoS One. 2022 Jul 26;17(7):e0270474. doi: 10.1371/journal.pone.0270474 (PMC9321764; doi:10.1371/journal.pone.0270474)
Supplement: S5 Table — (DOCX) [file pone.0270474.s005.docx]

**S5 Table.** Stable isotope (δ^13^Cˈ, δ^15^N, δ^18^O, δD) mean and range values for classified life history types of Broad Whitefish (*Coregonus nasus*) caught in the Colville River, AK, USA.

All stable isotope values are expressed in per mil (‰) relative to international standards.
